# The genome sequence of the alder spittlebug, *Aphrophora alni *(Fallén, 1805)

**DOI:** 10.12688/wellcomeopenres.23248.1

**Published:** 2024-10-29

**Authors:** Andy Griffiths, Stephen Moran, Liam M. Crowley, University of Oxford and Wytham Woods Genome Acquisition Lab, Wellcome Sanger Institute Tree of Life Management, Samples and Laboratory team, Wellcome Sanger Institute Scientific Operations: Sequencing Operations, Wellcome Sanger Institute Tree of Life Core Informatics team, Tree of Life Core Informatics collective, Darwin Tree of Life Consortium

**Affiliations:** 1 Wellcome Sanger Institute, Hinxton, England, UK; 2 Royal Botanic Garden Edinburgh, Edinburgh, Scotland, UK; 3 Highland Biological Recording Group, Inverness, Scotland, UK; 4 University of Oxford, Oxford, England, UK

**Keywords:** Aphrophora alni, alder spittlebug, genome sequence, chromosomal, Hemiptera

## Abstract

We present a genome assembly from an individual male *Aphrophora alni* (the alder spittlebug; Arthropoda; Insecta; Hemiptera; Aphrophoridae). The genome sequence has a total length of 1,781.50 megabases. Most of the assembly is scaffolded into 15 chromosomal pseudomolecules, including the X sex chromosome. The mitochondrial genome has also been assembled and is 27.61 kilobases in length. Gene annotation of this assembly on Ensembl identified 13,940 protein-coding genes.

## Species taxonomy

Eukaryota; Opisthokonta; Metazoa; Eumetazoa; Bilateria; Protostomia; Ecdysozoa; Panarthropoda; Arthropoda; Mandibulata; Pancrustacea; Hexapoda; Insecta; Dicondylia; Pterygota; Neoptera; Paraneoptera; Hemiptera; Auchenorrhyncha; Cicadomorpha; Cercopoidea; Aphrophoridae; *Aphrophora*; *Aphrophora alni* (Fallén, 1805) (NCBI:txid295201).

## Background

The alder spittlebug *Aphrophora alni*, family Aphrophoridae, is a species within the superfamily Cercopoidea, commonly known as the froghoppers. The genus *Aphrophora* is recognised by its larger size relative to other froghoppers, and a distinct, pale keel on the midline of the head and pronotum. *A. alni* is found across diverse habitats including woodlands, large gardens, and open habitats. Despite a nomenclatural link with alder, *Alnus glutinosa*, it is associated with a wide range of trees and shrubs ( Le Quesne, 1965). It is distributed across Europe from Ireland to Russia and from the Mediterranean in the south to north of the Arctic Circle. Odd records are scattered across Russia to the Pacific ( GBIF Secretariat, 2024), and it has also been introduced to North America ( Simpson *et al.*, 2022).

*A. alni* is dark brown, usually with two pale patches along the costal margin of the forewings. Smooth, shiny forewings covered with black tubercles help distinguish *A. alni* from similar colour forms of *Philaenus spumarius* ( Stewart & Harkin, 2020) *. A. alni* is the most common and widespread of the four UK *Aphrophora* species ( Wilson *et al.*, 2015), and can be easily distinguished from them by its smaller size and the costal markings. *A. major* Uhler, 1896, *A. pectoralis* Matsumara, 1903 and *A. salicina* (Goeze, 1778) are all in excess of 9 mm in length, whereas *A. alni* is usually 6–9 mm ( Biedermann & Niedringhaus, 2009).

The species has been recorded as a pest on *Alnus*, and may also be a vector of the bacterial plant pathogen *Xylella fastidiosa* ( European Food Safety Authority, 2013). Nymphs of the superfamily Cercopoidea are widely known as ‘spittlebugs’, named for the foam they produce, commonly called cuckoo spit. This foam has adhesive properties that are of value to biotechnology ( Hoch *et al.*, 2024), which may be further explored using the reference genome presented here.

This data note presents the first chromosomally complete genome sequence for *Aphrophora alni*, based on a male specimen from Beinn Eighe National Nature Reserve, Scotland, UK.

## Genome sequence report

**Figure 1.**
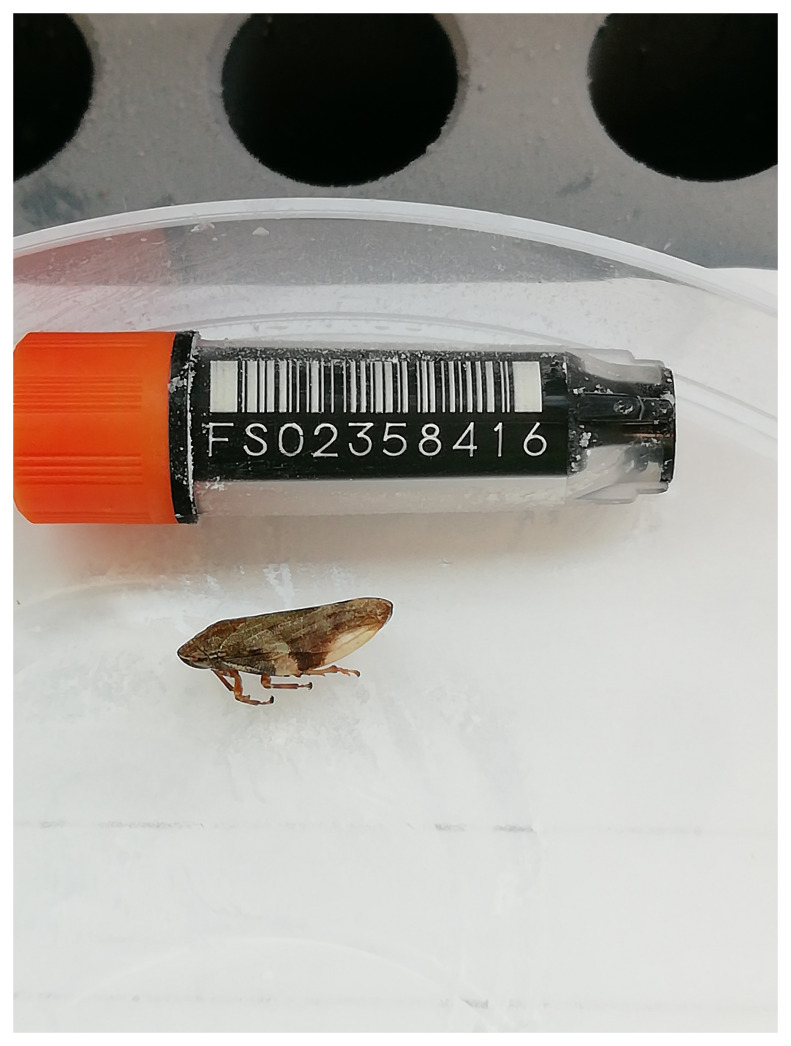
Photograph of *Aphrophora alni* by Barry Walter (not the specimen used for genome sequencing).

The genome of an adult male *Aphrophora alni* ( Figure 1) was sequenced using Pacific Biosciences single-molecule HiFi long reads, generating a total of 68.61 Gb (gigabases) from 7.34 million reads, giving an estimated 43-fold coverage, based on the GenomeScope size estimate. Primary assembly contigs were scaffolded with chromosome conformation Hi-C data, which produced 494.20 Gbp from 3,272.86 million reads, yielding an approximate coverage of 277-fold. Specimen and sequencing details are summarised in Table 1.

**Table 1.** Specimen and sequencing data for *Aphrophora alni*.

Project information			
**Study title**	*Aphrophora alni* (alder spittlebug)		
**Umbrella BioProject**	PRJEB65666		
**Species**	*Aphrophora alni*		
**BioSample**	SAMEA110180667		
**NCBI taxonomy ID**	295201		
Specimen information			
**Technology**	**ToLID**	**BioSample accession**	**Organism part**
**PacBio long read sequencing**	ihAphAlni3	SAMEA110180688	Whole organism
**Hi-C sequencing**	ihAphAlni1	SAMEA7520421	Whole organism
Sequencing information			
**Platform**	**Run accession**	**Read count**	**Base count (Gb)**
**Hi-C Illumina NovaSeq 6000**	ERR12035232	3.27e+09	494.2
**PacBio Revio**	ERR12015732	7.34e+06	68.61

Assembly errors were corrected by manual curation, including 159 missing joins or mis-joins and 13 haplotypic duplications. This reduced the assembly length by 0.9% and the scaffold number by 6.1%, and increased the scaffold N50 by 2.24%. The final assembly has a total length of 1,781.50 Mb in 1,000 sequence scaffolds with a scaffold N50 of 133.1 Mb ( Table 2). The total count of gaps in the scaffolds is 2,090.

**Table 2.** Genome assembly data for *Aphrophora alni*, ihAphAlni3.1.

Genome assembly		
Assembly name	ihAphAlni3.1	
Assembly accession	GCA_963513935.1	
*Accession of alternate haplotype*	*GCA_963513995.1*	
Span (Mb)	1,781.50	
Number of contigs	3,091	
Contig N50 length (Mb)	1.3	
Number of scaffolds	1,000	
Scaffold N50 length (Mb)	133.1	
Longest scaffold (Mb)	173.06	
Assembly metrics *		*Benchmark*
Consensus quality (QV)	55.9	*≥ 50*
*k*-mer completeness	99.99%	*≥ 95%*
BUSCO **	C:98.3%[S:96.5%,D:1.8%], F:0.8%,M:0.8%,n:2,510	*C ≥ 95%*
Percentage of assembly mapped to chromosomes	93.7%	*≥ 95%*
Sex chromosomes	XO	*localised homologous pairs*
Organelles	Mitochondrial genome: 27.61 kb	*complete single alleles*
Genome annotation of assembly GCA_963513935.1 at Ensembl		
Number of protein-coding genes	13,940	
Number of non-coding genes	8,797	
Number of gene transcripts	30,759	

*Assembly metric benchmarks are adapted from column VGP-2020 of “Table 1: Proposed standards and metrics for defining genome assembly quality” from Rhie *et al.* (2021). **BUSCO scores based on the hemiptera_odb10 BUSCO set using version 5.3.2. C = complete [S = single copy, D = duplicated], F = fragmented, M = missing, n = number of orthologues in comparison. A full set of BUSCO scores is available at https://blobtoolkit.genomehubs.org/view/CAUPSG01/dataset/CAUPSG01/busco.

**Figure 2.**
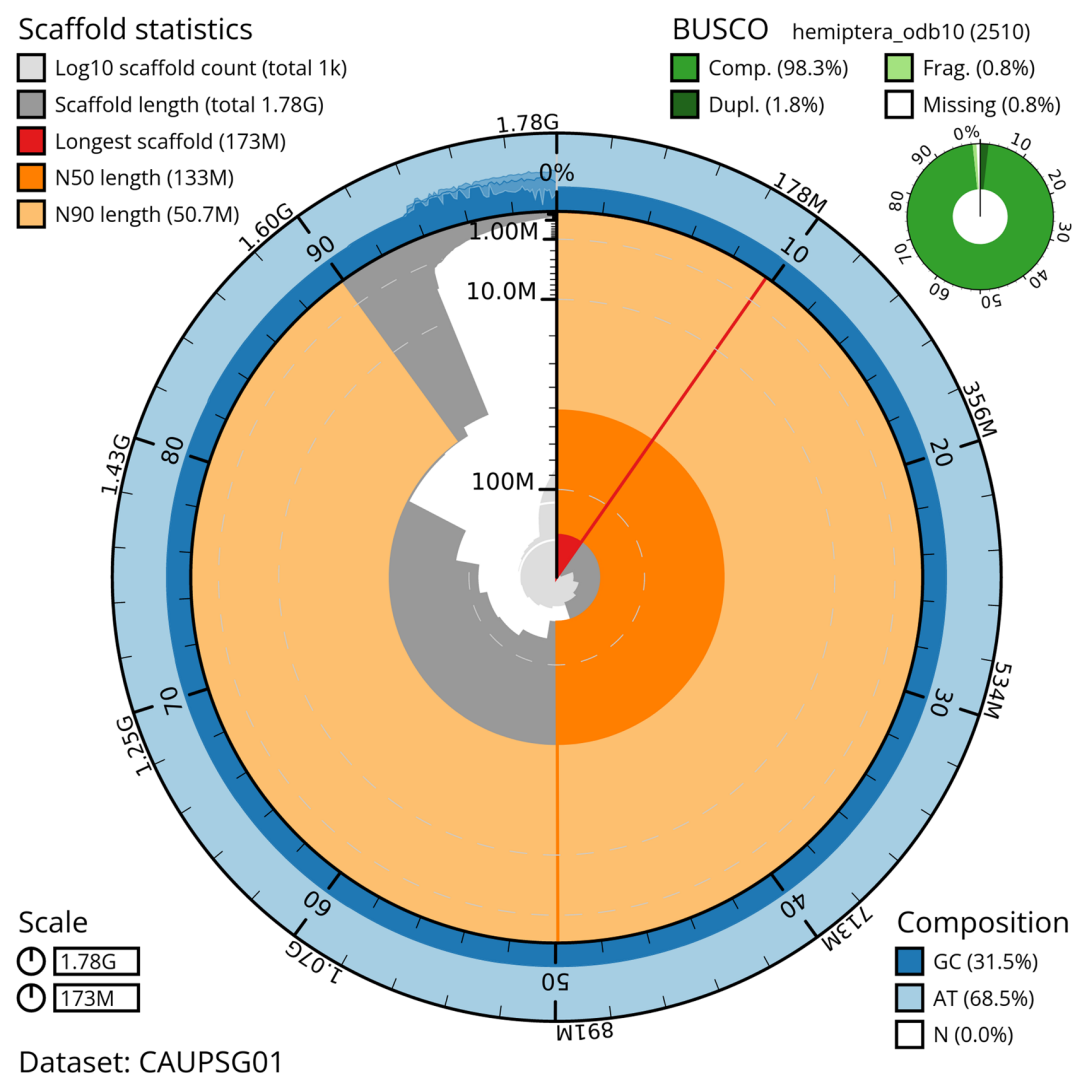
Genome assembly of *Aphrophora alni*, ihAphAlni3.1: metrics. The BlobToolKit snail plot shows N50 metrics and BUSCO gene completeness. The main plot is divided into 1,000 size-ordered bins around the circumference with each bin representing 0.1% of the 1,781,524,977 bp assembly. The distribution of scaffold lengths is shown in dark grey with the plot radius scaled to the longest scaffold present in the assembly (173,057,337 bp, shown in red). Orange and pale-orange arcs show the N50 and N90 scaffold lengths (133,089,614 and 50,706,419 bp), respectively. The pale grey spiral shows the cumulative scaffold count on a log scale with white scale lines showing successive orders of magnitude. The blue and pale-blue area around the outside of the plot shows the distribution of GC, AT and N percentages in the same bins as the inner plot. A summary of complete, fragmented, duplicated and missing BUSCO genes in the hemiptera_odb10 set is shown in the top right. An interactive version of this figure is available at https://blobtoolkit.genomehubs.org/view/CAUPSG01/dataset/CAUPSG01/snail.

**Figure 3.**
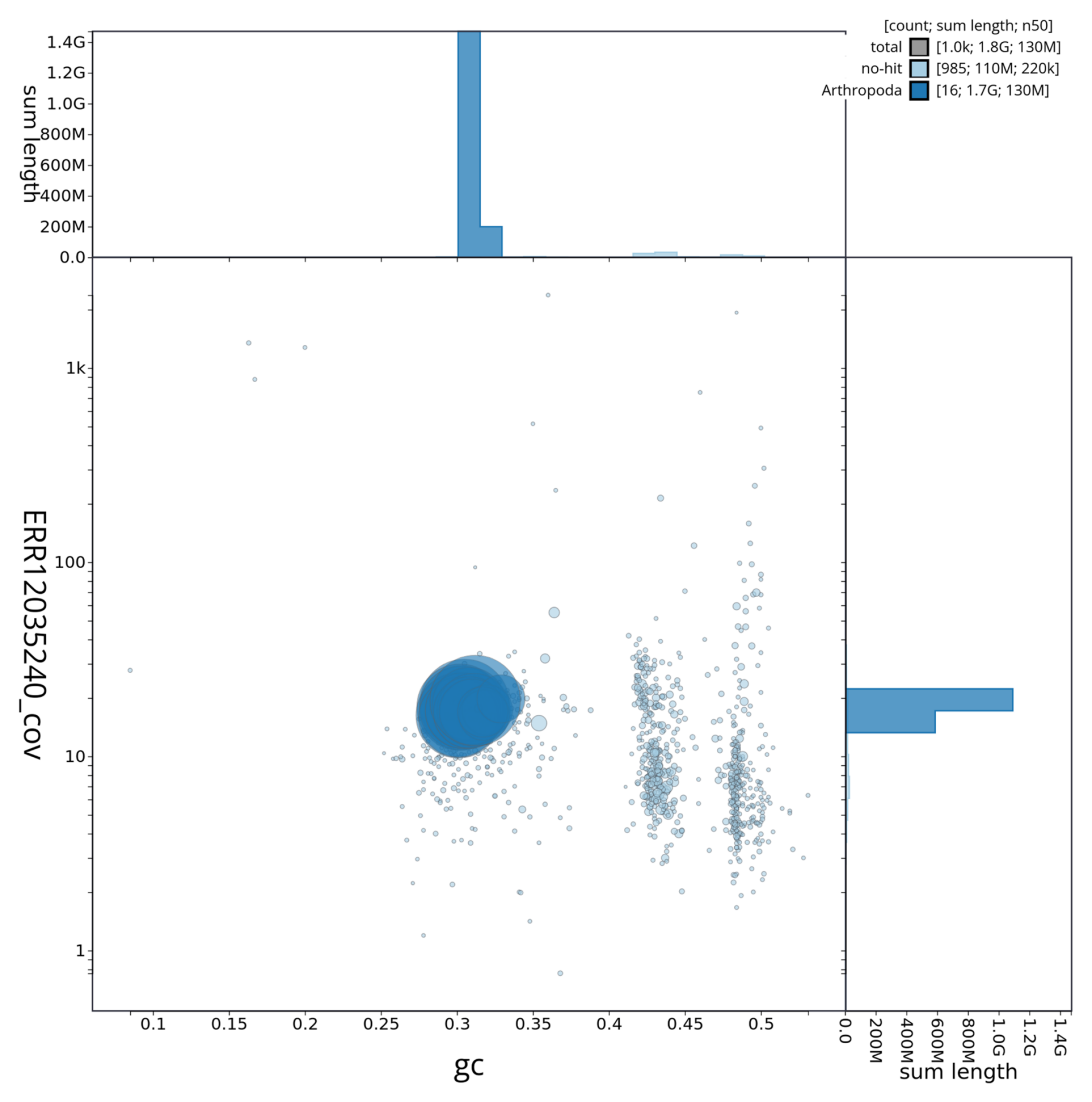
Genome assembly of *Aphrophora alni*, ihAphAlni3.1: Blob plot of base coverage against GC proportion for sequences in the assembly. Sequences are coloured by phylum. Circles are sized in proportion to sequence length. Histograms show the distribution of sequence length sum along each axis. An interactive version of this figure is available at https://blobtoolkit.genomehubs.org/view/CAUPSG01/dataset/CAUPSG01/blob.

**Figure 4.**
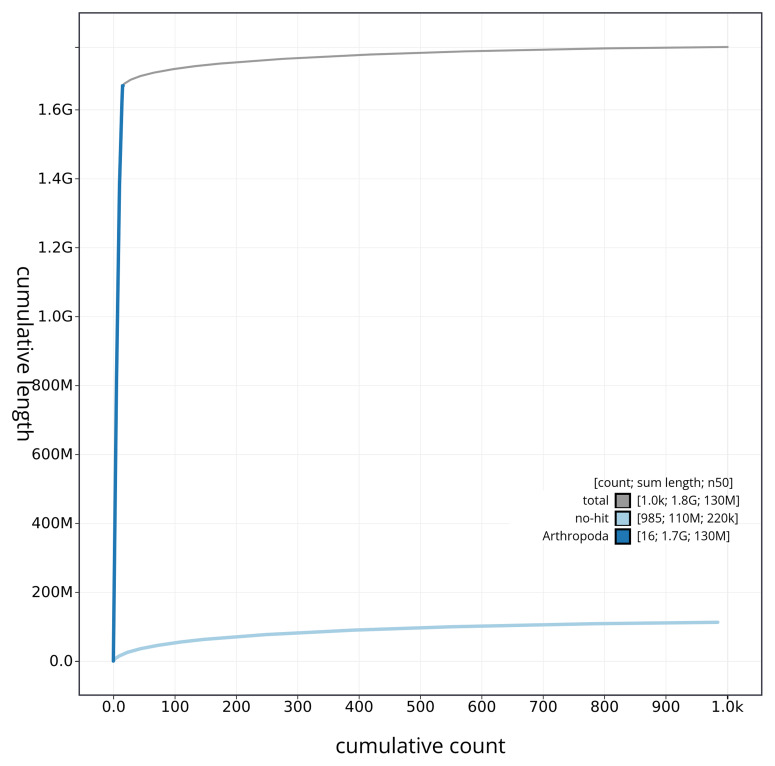
Genome assembly of *Aphrophora alni* ihAphAlni3.1: BlobToolKit cumulative sequence plot. The grey line shows cumulative length for all sequences. Coloured lines show cumulative lengths of sequences assigned to each phylum using the buscogenes taxrule. An interactive version of this figure is available at https://blobtoolkit.genomehubs.org/view/CAUPSG01/dataset/CAUPSG01/cumulative.

The snail plot in Figure 2 provides a summary of the assembly statistics, indicating the distribution of scaffold lengths and other assembly metrics. Figure 3 shows the distribution of scaffolds by GC proportion and coverage. Figure 4 presents a cumulative assembly plot, with separate curves representing different scaffold subsets assigned to various phyla, illustrating the completeness of the assembly.

**Figure 5.**
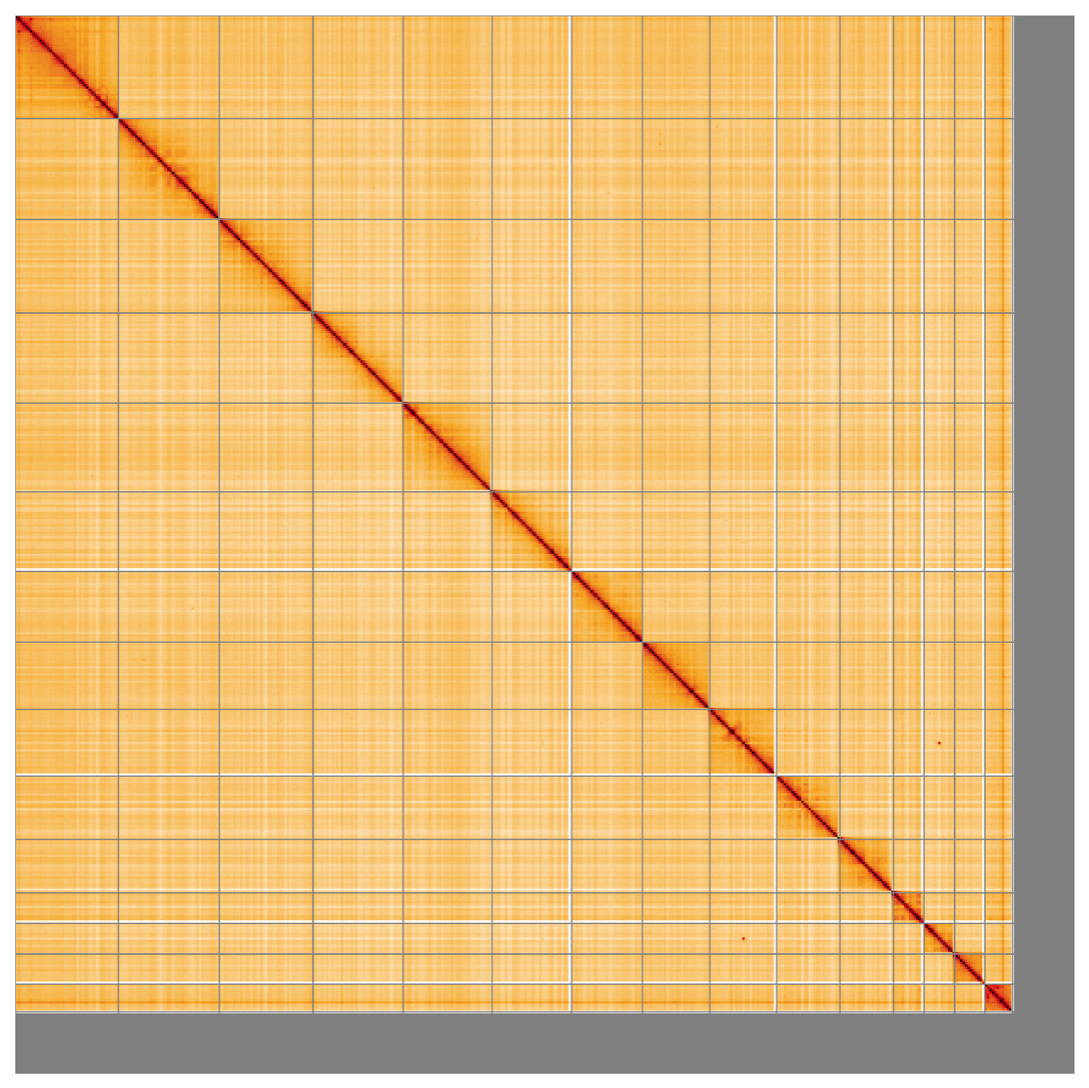
Genome assembly of *Aphrophora alni* ihAphAlni3.1: Hi-C contact map of the ihAphAlni3.1 assembly, visualised using HiGlass. Chromosomes are shown in order of size from left to right and top to bottom. An interactive version of this figure may be viewed at https://genome-note-higlass.tol.sanger.ac.uk/l/?d=GhUhMHyERMSIdeNqZ-unLQ.

Most of the assembly sequence (93.7%) was assigned to 15 chromosomal-level scaffolds, representing 14 autosomes and the X sex chromosome. During assembly curation it was noted that the specimen is an XO male. Chromosome-scale scaffolds confirmed by the Hi-C data are named in order of size ( Figure 5; Table 3).

**Table 3.** Chromosomal pseudomolecules in the genome assembly of *Aphrophora alni*, ihAphAlni3.

INSDC accession	Name	Length (Mb)	GC%
OY740766.1	1	168.43	30.5
OY740767.1	2	156.57	30.0
OY740768.1	3	151.51	30.0
OY740769.1	4	148.28	30.5
OY740770.1	5	133.09	30.0
OY740771.1	6	118.61	30.5
OY740772.1	7	112.84	31.0
OY740773.1	8	111.56	31.0
OY740774.1	9	106.13	31.0
OY740775.1	10	89.34	31.0
OY740776.1	11	51.23	32.0
OY740777.1	12	51.23	32.0
OY740778.1	13	50.71	31.5
OY740779.1	14	45.99	33.0
OY740765.1	X	173.06	31.0
OY740780.1	MT	0.03	20.0

While not fully phased, the assembly deposited is of one haplotype. Contigs corresponding to the second haplotype have also been deposited. The mitochondrial genome was also assembled and can be found as a contig within the multifasta file of the genome submission, and as a separate fasta file with accession OY740780.1.

The final assembly has a Quality Value (QV) of 55.9 and *k*-mer completeness of 99.99%. BUSCO (v5.3.2) analysis using the hemiptera_odb10 reference set ( *n* = 2,510). indicated a completeness score of 98.3% (single = 96.5%, duplicated = 1.8%).

Metadata for specimens, BOLD barcode results, spectra estimates, sequencing runs, contaminants and pre-curation assembly statistics are given at https://links.tol.sanger.ac.uk/species/295201.

## Genome annotation report

The *Aphrophora alni* genome assembly (GCA_963513935.1) was annotated at the European Bioinformatics Institute (EBI) on Ensembl Rapid Release. The resulting annotation includes 30,759 transcribed mRNAs from 13,940 protein-coding and 8,797 non-coding genes ( Table 2; https://rapid.ensembl.org/Aphrophora_alni_GCA_963513935.1/Info/Index). The average transcript length is 24,072.38. There are 1.35 coding transcripts per gene and 5.41 exons per transcript.

## Methods

### Sample acquisition

An adult specimen of *Aphrophora alni* (specimen ID SAN0001811, ToLID ihAphAlni3) was collected from Beinn Eighe National Nature Reserve, Scotland, UK (latitude 57.63, longitude –5.35) on 2021-08-04. The specimen was collected by Stephen Moran (Highland Biological Recording Group) and Andy Griffiths (Wellcome Sanger Institute), identified by Stephen Moran, and preserved by flash freezing.

The specimen used for Hi-C sequencing (specimen ID Ox000129, ToLID ihAphAlni1) was an adult specimen potted in Wytham Woods, Oxfordshire (biological vice-county Berkshire), UK (latitude 51.77, longitude –1.34) on 2019-08-07. The specimen was collected and identified by Liam Crowley (University of Oxford) and preserved on dry ice.

### Nucleic acid extraction

The workflow for high molecular weight (HMW) DNA extraction at the Wellcome Sanger Institute (WSI) Tree of Life Core Laboratory includes a sequence of core procedures: sample preparation and homogenisation, DNA extraction, fragmentation and purification. Detailed protocols are available on protocols.io ( Denton *et al.*, 2023b). The ihAphAlni3 sample was prepared for DNA extraction by weighing and dissecting it on dry ice ( Jay *et al.*, 2023), and tissue derived from the whole organism was homogenised using a PowerMasher II tissue disruptor ( Denton *et al.*, 2023a).

HMW DNA was extracted in the WSI Scientific Operations core using the Automated MagAttract v2 protocol ( Oatley *et al.*, 2023). The DNA was sheared into an average fragment size of 12–20 kb in a Megaruptor 3 system ( Bates *et al.*, 2023). Sheared DNA was purified by solid-phase reversible immobilisation, using AMPure PB beads to eliminate shorter fragments and concentrate the DNA ( Strickland *et al.*, 2023). The concentration of the sheared and purified DNA was assessed using a Nanodrop spectrophotometer and Qubit Fluorometer using the Qubit dsDNA High Sensitivity Assay kit. Fragment size distribution was evaluated by running the sample on the FemtoPulse system.

### Sequencing

Pacific Biosciences HiFi circular consensus DNA sequencing libraries were constructed according to the manufacturers’ instructions. DNA sequencing was performed by the Scientific Operations core at the WSI on a Pacific Biosciences Revio instrument.

Hi-C data were generated from the whole organism tissue of the ihAphAlni1 sample, using the Arima-HiC v2 kit. Frozen tissue (–80 °C) was fixed, and the DNA crosslinked using a TC buffer containing formaldehyde. The crosslinked DNA was then digested using a restriction enzyme master mix. The 5’-overhangs were then filled in and labelled with a biotinylated nucleotide and proximally ligated. The biotinylated DNA construct was fragmented to a fragment size of 400 to 600 bp using a Covaris E220 sonicator. The DNA was then enriched, barcoded, and amplified using the NEBNext Ultra II DNA Library Prep Kit, following manufacturers’ instructions. The Hi-C sequencing was performed using paired-end sequencing with a read length of 150 bp on an Illumina NovaSeq 6000 instrument.

### Genome assembly, curation and evaluation

***Assembly***

The HiFi reads were first assembled using Hifiasm ( Cheng *et al.*, 2021) with the --primary option. Haplotypic duplications were identified and removed using purge_dups ( Guan *et al.*, 2020). The Hi-C reads were mapped to the primary contigs using bwa-mem2 ( Vasimuddin *et al.*, 2019). The contigs were further scaffolded using the provided Hi-C data ( Rao *et al.*, 2014) in YaHS ( Zhou *et al.*, 2023) using the --break option. The scaffolded assemblies were evaluated using Gfastats ( Formenti *et al.*, 2022), BUSCO ( Manni *et al.*, 2021) and MERQURY.FK ( Rhie *et al.*, 2020).

The mitochondrial genome was assembled using MitoHiFi ( Uliano-Silva *et al.*, 2023), which runs MitoFinder ( Allio *et al.*, 2020) and uses these annotations to select the final mitochondrial contig and to ensure the general quality of the sequence.

***Assembly curation***

The assembly was decontaminated using the Assembly Screen for Cobionts and Contaminants (ASCC) pipeline (article in preparation). Flat files and maps used in curation were generated in TreeVal ( Pointon *et al.*, 2023). Manual curation was primarily conducted using PretextView ( Harry, 2022), with additional insights provided by JBrowse2 ( Diesh *et al.*, 2023) and HiGlass ( Kerpedjiev *et al.*, 2018). Scaffolds were visually inspected and corrected as described by Howe *et al*. (2021). Any identified contamination, missed joins, and mis-joins were corrected, and duplicate sequences were tagged and removed. The sex chromosome was identified based on read coverage statistics. The entire process is documented at https://gitlab.com/wtsi-grit/rapid-curation (article in preparation).

***Evaluation of the final assembly***

A Hi-C map for the final assembly was produced using bwa-mem2 ( Vasimuddin *et al.*, 2019) in the Cooler file format ( Abdennur & Mirny, 2020). To assess the assembly metrics, the *k*-mer completeness and QV consensus quality values were calculated in Merqury ( Rhie *et al.*, 2020). This work was done using the “sanger-tol/readmapping” ( Surana *et al.*, 2023a) and “sanger-tol/genomenote” ( Surana *et al.*, 2023b) pipelines. The genome readmapping pipelines were developed using the nf-core tooling ( Ewels *et al.*, 2020), use MultiQC ( Ewels *et al.*, 2016), and make extensive use of the Conda package manager, the Bioconda initiative ( Grüning *et al.*, 2018), the Biocontainers infrastructure ( da Veiga Leprevost *et al.*, 2017), and the Docker ( Merkel, 2014) and Singularity ( Kurtzer *et al.*, 2017) containerisation solutions. The genome was also analysed within the BlobToolKit environment ( Challis *et al.*, 2020) and BUSCO scores ( Manni *et al.*, 2021) were calculated.

Table 4 contains a list of relevant software tool versions and sources.

**Table 4.** Software tools: versions and sources.

Software tool	Version	Source
BlobToolKit	4.2.1	https://github.com/blobtoolkit/blobtoolkit
BUSCO	5.3.2	https://gitlab.com/ezlab/busco
bwa-mem2	2.2.1	https://github.com/bwa-mem2/bwa-mem2
Cooler	0.8.11	https://github.com/open2c/cooler
Gfastats	1.3.6	https://github.com/vgl-hub/gfastats
Hifiasm	0.16.1	https://github.com/chhylp123/hifiasm
HiGlass	1.11.6	https://github.com/higlass/higlass
Merqury.FK	d00d98157618f4e8d1a91 90026b19b471055b22e	https://github.com/thegenemyers/MERQURY.FK
MitoHiFi	3.2	https://github.com/marcelauliano/MitoHiFi
PretextView	0.2	https://github.com/wtsi-hpag/PretextView
purge_dups	1.2.3	https://github.com/dfguan/purge_dups
sanger-tol/genomenote	v1.0	https://github.com/sanger-tol/genomenote
sanger-tol/readmapping	1.1.0	https://github.com/sanger-tol/readmapping/tree/1.1.0
Singularity	3.9.0	https://github.com/sylabs/singularity
YaHS	1.1a.2	https://github.com/c-zhou/yahs

### Genome annotation

The Ensembl Genebuild annotation system ( Aken *et al.*, 2016) was used to generate annotation for the *Aphrophora alni* assembly (GCA_963513935.1) in Ensembl Rapid Release at the EBI. Annotation was created primarily through alignment of transcriptomic data to the genome, with gap filling via protein-to-genome alignments of a select set of proteins from UniProt ( UniProt Consortium, 2019).

### Wellcome Sanger Institute – Legal and Governance

The materials that have contributed to this genome note have been supplied by a Darwin Tree of Life Partner. The submission of materials by a Darwin Tree of Life Partner is subject to the **‘Darwin Tree of Life Project Sampling Code of Practice’**, which can be found in full on the Darwin Tree of Life website here. By agreeing with and signing up to the Sampling Code of Practice, the Darwin Tree of Life Partner agrees they will meet the legal and ethical requirements and standards set out within this document in respect of all samples acquired for, and supplied to, the Darwin Tree of Life Project.

Further, the Wellcome Sanger Institute employs a process whereby due diligence is carried out proportionate to the nature of the materials themselves, and the circumstances under which they have been/are to be collected and provided for use. The purpose of this is to address and mitigate any potential legal and/or ethical implications of receipt and use of the materials as part of the research project, and to ensure that in doing so we align with best practice wherever possible. The overarching areas of consideration are:

•      Ethical review of provenance and sourcing of the material

•      Legality of collection, transfer and use (national and international)

Each transfer of samples is further undertaken according to a Research Collaboration Agreement or Material Transfer Agreement entered into by the Darwin Tree of Life Partner, Genome Research Limited (operating as the Wellcome Sanger Institute), and in some circumstances other Darwin Tree of Life collaborators.

## Data Availability

European Nucleotide Archive: Aphrophora alni (alder spittlebug). Accession number PRJEB65666; https://identifiers.org/ena.embl/PRJEB65666 ( Wellcome Sanger Institute, 2023). The genome sequence is released openly for reuse. The *Aphrophora alni* genome sequencing initiative is part of the Darwin Tree of Life (DToL) project. All raw sequence data and the assembly have been deposited in INSDC databases. Raw data and assembly accession identifiers are reported in Table 1 and Table 2.

## Author information

Members of the University of Oxford and Wytham Woods Genome Acquisition Lab are listed here: https://doi.org/10.5281/zenodo.12157525.

Members of the Wellcome Sanger Institute Tree of Life Management, Samples and Laboratory team are listed here: https://doi.org/10.5281/zenodo.12162482.

Members of Wellcome Sanger Institute Scientific Operations: Sequencing Operations are listed here: https://doi.org/10.5281/zenodo.12165051.

Members of the Wellcome Sanger Institute Tree of Life Core Informatics team are listed here: https://doi.org/10.5281/zenodo.12160324.

Members of the Tree of Life Core Informatics collective are listed here: https://doi.org/10.5281/zenodo.12205391.

Members of the Darwin Tree of Life Consortium are listed here: https://doi.org/10.5281/zenodo.4783558.
