# Supplementary material for: The genome sequence of the alder spittlebug, Aphrophora alni (Fallén, 1805)
Source: Wellcome Open Res. 2024 Oct 29;9:632. [Version 1] doi: 10.12688/wellcomeopenres.23248.1 (PMC11579584; doi:10.12688/wellcomeopenres.23248.1)
Supplement: Peer review history

# Reviewer report: Reviewer response for version 1

**DOI:** 10.21956/wellcomeopenres.25620.r109981

**Published:** 2024-11-20

**Authors:** Li Lim

**Affiliations:** 1 Universiti Sains Malaysia, George Town, Malaysia

The manuscript presents a significant contribution to the field by offering the chromosomally complete genome assembly of *Aphrophora alni*, the alder spittlebug. The study is well-structured, and the methodologies employed, including the integration of PacBio HiFi and Hi-C data, are robust and comprehensive. The detailed curation and annotation processes provide confidence in the quality and completeness of the assembly.

 Specific comment:

 Figure 1 should be refined. The insect photograph should be taken under a dissecting microscope and include an appropriate scale bar.

Are sufficient details of methods and materials provided to allow replication by others?

Yes

Is the rationale for creating the dataset(s) clearly described?

Yes

Are the datasets clearly presented in a useable and accessible format?

Yes

Are the protocols appropriate and is the work technically sound?

Yes

Reviewer Expertise:

Molecular biology, entomology, plant pathology

I confirm that I have read this submission and believe that I have an appropriate level of expertise to confirm that it is of an acceptable scientific standard.

# Reviewer report: Reviewer response for version 1

**DOI:** 10.21956/wellcomeopenres.25620.r109973

**Published:** 2024-11-12

**Authors:** Qianquan Chen

**Affiliations:** 1 Guizhou Normal University, Guiyang, Guizhou, China

*Aphrophora alni* distributes across Europe. It has been recorded as a pest on *Alnus*, and may be a vector of the bacterial plant pathogen *Xylella fastidiosa*. Nymphs of the superfamily Cercopoidea are widely known as ‘spittlebugs’ for foam. The foam has adhesive properties that are of value to biotechnology. The genome sequence can contribute to the study of the *A. alni*. I advise authors to use a new photo which can clearly show the morphological characteristics of the *A. alni*.

Are sufficient details of methods and materials provided to allow replication by others?

Yes

Is the rationale for creating the dataset(s) clearly described?

Yes

Are the datasets clearly presented in a useable and accessible format?

Yes

Are the protocols appropriate and is the work technically sound?

Yes

Reviewer Expertise:

Eco-genomics

I confirm that I have read this submission and believe that I have an appropriate level of expertise to confirm that it is of an acceptable scientific standard.
